# Complete Genome Sequence of *Marinobacter* sp. BSs20148

**DOI:** 10.1128/genomeA.00236-13

**Published:** 2013-05-16

**Authors:** Lai Song, Lufeng Ren, Xingang Li, Dan Yu, Yong Yu, Xumin Wang, Guiming Liu

**Affiliations:** CAS Key Laboratory of Genome Sciences and Information, Beijing Institute of Genomics, Chinese Academy of Sciences, Beijing, Chinaa;; Polar Research Institute of China, Shanghai , Chinab;; Graduate University of Chinese Academy of Sciences, Beijing, Chinac

## Abstract

*Marinobacter* sp. BSs20148 was isolated from marine sediment collected from the Arctic Ocean at a water depth of 3,800 m. Here we report the complete genome sequence of *Marinobacter* sp. BSs20148. This genomic information will facilitate the study of the physiological metabolism, ecological roles, and evolution of the *Marinobacter* species.

## GENOME ANNOUNCEMENT

The genus *Marinobacter* was proposed by Gauthier et al. (1) to accommodate Gram-negative, aerobic, moderately halophilic gammaproteobacteria that use several hydrocarbons as sole sources of carbon and energy. Hitherto the genus comprised 18 species, including those recently added to the genus, such as *Marinobacter algicola* (2), *Marinobacter gudaonensis* (3), *Marinobacter salsuginis* (4), and *Marinobacter vinifirmus* (5). *Marinobacter* sp. BSs20148 was isolated from deep-sea sediment (at a water depth of 3,800 m) of the Arctic Ocean (149°06′55′′W, 78°23′14′′N). Phylogenetic analyses based on 16S rRNA gene sequences revealed that *Marinobacter* sp. BSs20148 has the highest sequence similarity to *Marinobacter* sp. 20041^T^, a novel species isolated from sea-ice of the Canadian Basin (6).

The genome sequence of *Marinobacter* sp. BSs20148 was sequenced with the 454 GS-FLX platform. The library generated 184,415 reads totaling 69,606,604 nucleotide bases, with an average read length of 377 and about 17-fold coverage of the genome. *De novo* assemblies were performed using Roche Newbler version 2.6, resulting in 37 large contigs. Gaps were closed by primer walking, long-distance PCR and optimized multiplex PCR (7). Sequences were assembled and edited using Phred/Phrap/Consed software (8). Open reading frames (ORFs) were predicted with Glimmer 3.02 (9). Putative ribosomal binding sites were identified with RBSfinder (10). tRNAs and rRNAs were predicted using tRNAscan-SE (11) and RNAmmer (12), respectively. Functional annotations were performed by a search against the NCBI nr, Swiss-Prot (13), COG (14), KEGG (15), and InterProScan (16) databases.

The chromosome was 4,063,864 bp in length with a GC content of 54.13%, containing 3,952 protein-encoding genes with an average length of 914 bases, 49 tRNA, and 3 rRNA-encoding operons. Approximately 62.98% of all coding sequences (a total of 2,489) were assigned to 21 COG function categories, and 1,815 CDSs, involved in 154 metabolic pathways, were annotated into 1,729 KEGG orthology groups by using KAAS (17). The genomic information will facilitate the study of physiology, adaptation, and evolution of the *Marinobacter* species.

### Nucleotide sequence accession number.

The genome data have been deposited in GenBank under the accession number CP003735.
